# A comprehensive profile of genomic variations in the SARS-CoV-2 isolates from the state of Telangana, India

**DOI:** 10.1099/jgv.0.001562

**Published:** 2021-02-15

**Authors:** Asmita Gupta, Radhakrishnan Sabarinathan, Pratyusha Bala, Vinay Donipadi, Divya Vashisht, Madhumohan Rao Katika, Manohar Kandakatla, Debashis Mitra, Ashwin Dalal, Murali Dharan Bashyam

**Affiliations:** ^1^​ Centre for DNA Fingerprinting and Diagnostics, Hyderabad, India; ^2^​ National Centre for Biological Sciences, Tata Institute of Fundamental Research, Bangalore, India; ^3^​ Nizam’s Institute of Medical Sciences, Hyderabad, India; ^‡^​ Present address: National Centre for Cell Science, Pune, India

**Keywords:** SARS-CoV-2, Genomic variants, Phylodynamic analysis

## Abstract

The novel severe acute respiratory syndrome coronavirus 2 (SARS-CoV-2) causing COVID-19 has rapidly turned into a pandemic, infecting millions and causing 1 157 509 (as of 27 October 2020) deaths across the globe. In addition to studying the mode of transmission and evasion of host immune system, analysing the viral mutational landscape constitutes an area under active research. The latter is expected to impart knowledge on the emergence of different clades, subclades, viral protein functions and protein–protein and protein–RNA interactions during replication/transcription cycle of virus and response to host immune checkpoints. In this study, we have attempted to bring forth the viral genomic variants defining the major clade(s) as identified from samples collected from the state of Telangana, India. We further report a comprehensive draft of all genomic variations (including unique mutations) present in SARS-CoV-2 strain in the state of Telangana. Our results reveal the presence of two mutually exclusive subgroups defined by specific variants within the dominant clade present in the population. This work attempts to bridge the critical gap regarding the genomic landscape and associate mutations in SARS-CoV-2 from a highly infected southern region of India, which was lacking to date.

## Introduction

The outbreak of COVID-19 caused by the severe acute respiratory syndrome coronavirus 2 (SARS-CoV-2, also called novel coronavirus 2019-nCoV) in the Hubei province of China during late December 2019, has since transformed into a severe pandemic, spreading across more than 200 countries. As of 27 October 2020, 1 157 509 deaths have been reported worldwide, as per recent statistics released by the World Health Organization (WHO) (https://covid19.who.int/, 2020). SARS-CoV-2 belongs to the subfamily *Orthocoronavirinae* of the *Coronaviridae* family, classified under the order *Nidovirales* [1]. After host entry, the 29.9 kb positive-sense, single-stranded, unsegmented RNA genome of this virus is immediately translated to yield 15 non-structural proteins (nsps, 1–15). These nsps subsequently form multi-protein complexes to execute genomic replication and transcription [2, 3] that produce a genomic RNA template along with nine subgenomic RNAs. The latter are translated to form major structural proteins viz. spike (S), envelope (E), nucleocapsid (N) and membrane (M) proteins [4, 5]. The SARS-CoV-2 virus uses the spike (S) protein to bind to the angiotensin converting enzyme 2 (hACE2) present on human cells and gain host entry.

Outbreaks caused by members of the *Coronaviridae* family including severe acute respiratory syndrome (caused by SARS-CoV) in 2003, Middle East respiratory syndrome (caused by MERS-CoV) in 2012, and currently COVID-19 (caused by SARS-CoV-2), have frequently crossed inter-species barriers to cause zoonotic infections with variable infectivity and transmission rates and case-to-fatality ratios [6]. The genome of SARS-CoV-2 has been found to share a high degree of sequence similarity with SARS-CoV (>80 %) and a moderate sequence similarity with MERS-CoV (>50 %) [7, 8]. The basic reproductive number (R_0_) (a measure of virus transmissibility), as estimated by WHO for SARS-CoV-2 ranges from 2 to 2.5 [9, 10], is significantly higher than that of SARS-CoV (R_0_ – 1.7–1.9) and MERS-CoV (<1), reflective of its massive spread across the globe. The severity of the pandemic has demanded a concerted effort to comprehensively study the actively changing mutational landscape of the virus across multiple demographic locations and identify potential individual variants or variant clusters which might play a critical role in its spread and transmission dynamics.

India has been witnessing a massive upsurge in the total number of confirmed infections (7 946 429 cases, as of 27 October 2020, https://covid19.who.int/), making the country the second worst hit nation due to COVID-19. This has been accompanied by a rise in the number of per day fatalities due to infections (119 502 total deaths). The state of Telangana, located in south-central India, has particularly seen a high rate of infection and there appears to have been a sharp spike in the number of cases beginning from the second half of April 2020 (as of 27 October 2020, the tally stands at 214 917 confirmed cases with 1319 deaths).

In this study, we aimed at identifying the dominant viral lineages present in samples collected in Telangana, among all identified lineages of SARS-CoV-2. We further endeavoured to draft a comprehensive mutational landscape of the viral genome. Towards this end, we applied next-generation sequencing to determine the complete sequence of 210 SARS-CoV-2 RNA samples. From a combined phylodynamic and mutational analysis, we reveal the 20B clade to be the dominant viral lineage circulating in Telangana. We further show the presence of a few recurrent, novel mutations that result in two relatively mutually exclusive groups within the dominant 20B clade.

## Methods

### Sample collection and processing

The Centre for DNA Fingerprinting and Diagnostics (CDFD), Hyderabad, initiated reverse transcription-PCR (RT-PCR) based diagnostics for COVID-19 infection after approvals from Secretary, Department of Biotechnology (DBT), Government of India, Indian Council of Medical Research (ICMR) nominated nodal officer in Hyderabad, Telangana as well as from the Telangana state government. The work was initiated following approvals from the Institutional Bioethics committee and Biosafety committee. The samples were obtained as nasopharyngeal swabs collected in Viral Transport Medium from patients with symptoms suggestive of COVID-19 as well as asymptomatic primary contacts of affected cases from different parts of Telangana. The samples were transported to CDFD within 24 h while maintaining a cold chain. Total RNA was isolated using the RNA isolation kit as per manufacturer’s instructions (QIAmp Viral RNA Mini Kit; Cat. No. 52906; Qiagen, Hilden, Germany). Each RNA sample was subjected to RT-PCR for multiple viral genes [including E-gene and RNA-dependent RNA polymerase (RDRP) gene] using the LabGun COVID-19 assay (Cat. No. CV9017B; LabGenomics, Republic of Korea) or the Allplex 2019-nCoV Assay (Cat. No. RP10250X, Seegene, Republic of Korea). The samples that tested positive in RT-PCR analysis were included for viral genome sequencing. Since RdRp consistently provided more robust amplification than E-gene and is a SARS-CoV-2 specific gene (unlike E-gene, which is specific for all respiratory coronaviruses), we considered Ct (threshold cycle) values of RdRp alone for analysis. Samples exhibiting a Ct value greater than 10 and less than 35 were chosen for sequencing.

### Sequencing protocol

Sequencing of SARS-CoV-2 RNA samples was performed using the protocol described earlier (nCoV-2019 sequencing protocol; https://dx.doi.org/10.17504/protocols.io.bdp7i5rn) with slight modifications. Briefly, RNA isolated from nasopharyngeal swabs was reverse transcribed using random primer mix (New England Biolabs, MA, USA), and Superscript-IV (Thermofisher Scientific, MA, USA). The resulting cDNA was subjected to a three-step multiplex PCR using nCoV-2019/V3 primer pools (Eurofins, India) 1, 2 (nCoV-2019 sequencing protocol; https://dx.doi.org/10.17504/protocols.io.bdp7i5rn).

Amplicons 17 (from pool 1) and 18, 28 and 64 (all from pool 2) failed to amplify at the recommended annealing temperature (Ta) of 65 °C; these were separately amplified at a reduced Ta of 61 °C. The ∼400 bp amplicons thus obtained in the pools were combined (pool 1 and 2 : 25 µl each and pool 3 : 2 µl), purified using Agencourt AMPure XP beads (Beckman Coulter, CA, USA) and eluted in 45 µl elution buffer (Qiagen, Hilden, Germany). DNA libraries for Illumina sequencing were prepared using the NEB Next Ultra II DNA Library Prep Kit for Illumina (New England Biolabs, MA, USA), according to the manufacturer’s protocol. Paired-end sequencing (2×250 bp) was performed on the Miseq FGx (Illumina, CA, USA) with a targeted depth of 0.5 million reads per sample (∼4000× coverage). Libraries for Nanopore sequencing were prepared using the Ligation sequencing kit (LSK-109; Oxford Nanopore Technologies, London, UK). Barcoded libraries were pooled (12–24 samples each) and sequenced on a MinION flow cell in GridION (Oxford Nanopore Technologies, London, UK). Sequencing was performed with a targeted depth of 0.1 million reads per sample (up to 24 h).

### Mutation analysis

The dataset comprised a total of 210 unique strains sequenced using the aforementioned protocol. All raw fastq files from Illumina platform (123 samples) were checked for overall sequencing quality, the presence of adapters and bad quality reads using FastQC and Fastp [11]. The adapter sequences were trimmed using the 'Trim Galore' tool, a wrapper script provided by Cutadapt [12]. The filtered reads were aligned to the reference strain NC_045512.1, severe acute respiratory syndrome coronavirus 2 isolate Wuhan-Hu-1, using the bwa-mem [13] algorithm with default parameters. Mapping quality was assessed using samtools [14] and BAMStats. Post alignment, the reads were filtered, sorted and indexed using samtools, and any primer sequences were masked using iVar [15]. Subsequent mutation calling and generation of consensus sequence was performed using samtools, mpileup and iVar (https://github.com/connor-lab/ncov2019-artic-nf/). The resulting VCF files were annotated using snpEff [16]. For processing the nanopore data (95 samples), we followed the protocol suggested by the ARTIC pipeline (https://github.com/artic-network/fieldbioinformatics , https://github.com/connor-lab/ncov2019-artic-nf/). Since, eight samples were subjected to sequencing by both the Illumina and Nanopore pipeline, subsequent downstream analysis included only the unique sequence where quality was better either with the Illumina or Nanopore platform. A schematic describing the entire workflow is shown in Fig. S1 (available in the online version of this article). Before analysing the obtained calls, we filtered all the problematic sites prone to errors by multiple sources as recommended earlier (https://virological.org/t/issues-with-sars-cov-2-sequencing-data/473).

### Mutation distance and correlation matrix

A binary matrix was constructed for each sample, which was processed by Nextstrain (https://github.com/nextstrain/ncov) indicating whether a mutation of interest was present or absent. Pairwise Pearson correlation coefficients were estimated for this binary matrix. Further, a distance matrix was also calculated, which was subsequently used for hierarchical clustering of all high-frequency mutations identified. All analyses were performed using custom R scripts.

### Phylogenetic analysis

The consensus fasta files generated for both Illumina and Nanopore data were subjected to phylogenetic analysis using the Nextstrain pipeline with recommended default criteria for filtering, multiple sequence alignment (MSA) and nucleotide substitution calculations. To briefly summarize the workflow of the pipeline, all the consensus sequences having length <27 000 and N’s >5 % were filtered out. Three samples were removed from further analysis as their sequence data included N’s>5 %; thus all analyses were conducted on 207 individual patient viral genome sequences. A compendium of problematic sites as used earlier (https://virological.org/t/issues-with-sars-cov-2-sequencing-data/473), was also provided to mask those sites prior to MSA by MAFFT [17]. Following MSA, the workflow constructed a time-resolved phylogenetic tree using the maximum-likelihood-based method IQ-TREE [18], using the default nucleotide substitution model (‘GTR’ or general time reversible [19] implemented in IQ-TREE with 1000 bootstrap cycles). The resultant tree was pruned and internal nodes and ancestral traits were inferred from the dates of the sample collection using TreeTime [20]. The final tree in Newick format was then customized for visualization using iTol [21]. The nomenclature of Nextstrain-assigned phylogenetic clades is based on the naming scheme proposed by Rambaut *et al*. [22]. This scheme uses a year-letter nomenclature with numbers indicating the year of emergence of viral strain and letters A and B corresponding to the two root or reference sequences used for creating phylogenetic trees. Letter ‘A’ refers to the Wuhan/WH04/2020 (GISAID database accession – EPI_ISL_406801; GISAID-Global Initiative on Sharing All Influenza Data), while ‘B’ represents Wuhan-Hu-1 (GISAID accession – EPI_ISL_402125) sequence. These two sequences represent the earliest sampled strains of SARS-CoV-2, which were submitted to the GISAID database.

## Results

### General characteristics of the dataset

**Fig. 1.**
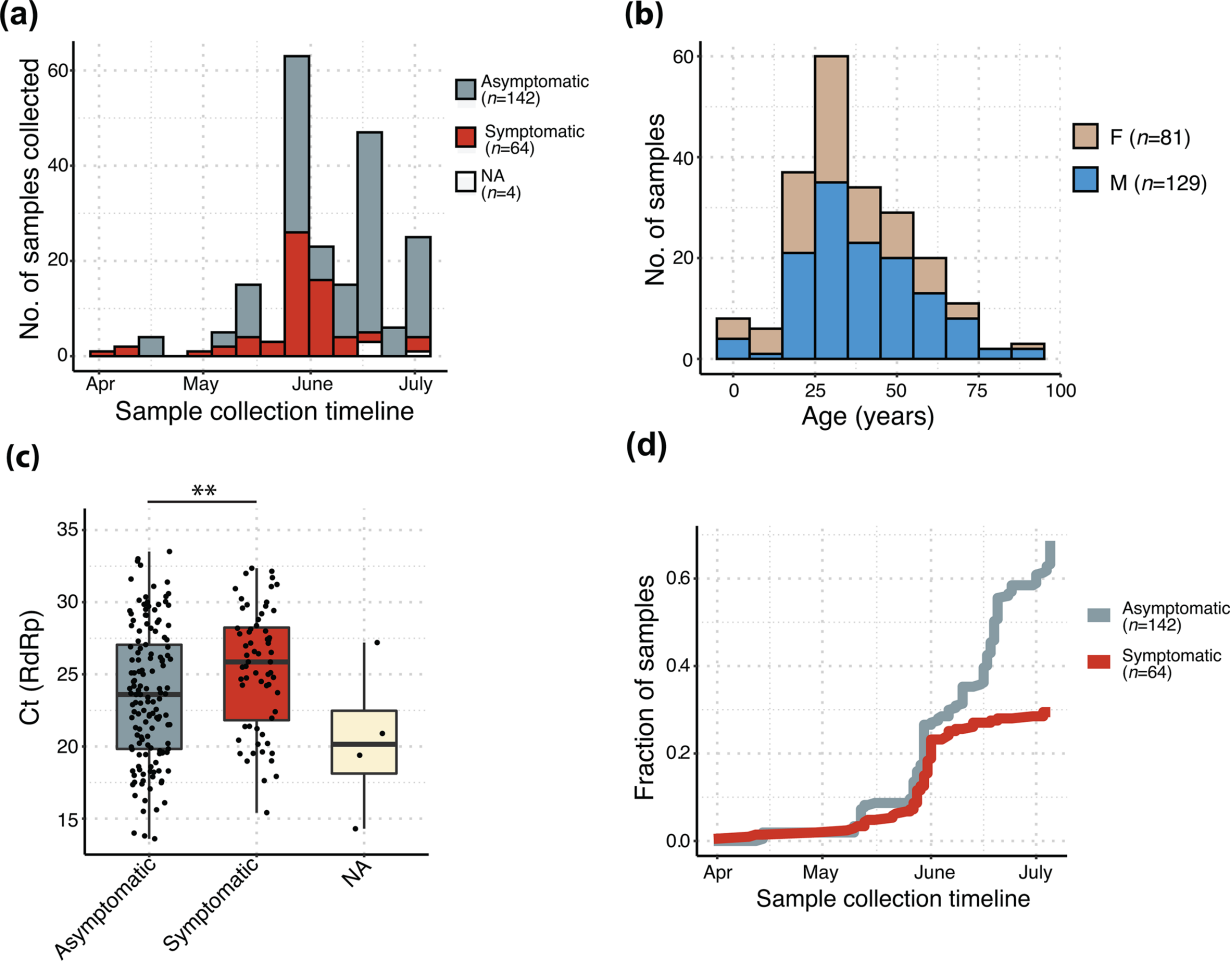
Characteristics of the dataset used in this study. (a) Distribution of sample collection timeline; (b) age and gender distribution across samples; (c) distribution of symptoms with respect to Ct values of RdRp gene (nsp12); Wilcoxon rank-sum test was used for calculating significance level (*, *P* <0.05); (d) cumulative increment of symptomatic and asymptomatic fraction of samples along the sample collection timeline.

Our dataset consisted of samples collected during late March to July 2020 and included a higher overall representation of asymptomatic cases (Fig. 1a). A majority of our samples belonged to age group between 15–62 years, with males (61 %) outnumbering females (39%) (Fig. 1b). Each bin in the age range was also populated by a higher number of males compared to females (Fig. 1b). We compared the distribution of sample symptoms with respect to Ct values, which acts as a proxy for viral load, and consequently indicates the likelihood of disease transmission. Our data indicated that symptomatic cases appeared to be associated with higher Ct values (thus lower viral load), compared to asymptomatic ones, which was unexpected (Fig. 1c). We observed a gradual reduction in the Ct values in samples, near the end of June 2020, implying that more recent samples seemed to carry a higher viral load than earlier samples (Fig. S2a). Despite the lack of samples after July, this trend could potentially explain the rise in viral transmission after this time period (https://covid19.who.int/table, https://www.mohfw.gov.in/). Furthermore, the distribution of Ct values with respect to age showed no clear correlation (Fig. S2b). The samples assigned as symptomatic mainly belonged to individuals coming from higher age groups (median age – 47.5 years) compared to the asymptomatic ones (median age – 30 years) (Fig. S2c). The dataset trends also indicated that there was a consistent rise in the fraction of samples showing asymptomatic behaviour, as calculated from a cumulative increment, starting from the end of May (Fig. 1d). The locale distribution of the samples in and around the district of Hyderabad is indicated in Fig. S2d.

### Phylodynamic epidemiological clustering of SARS-Cov2 strains

**Fig. 2.**
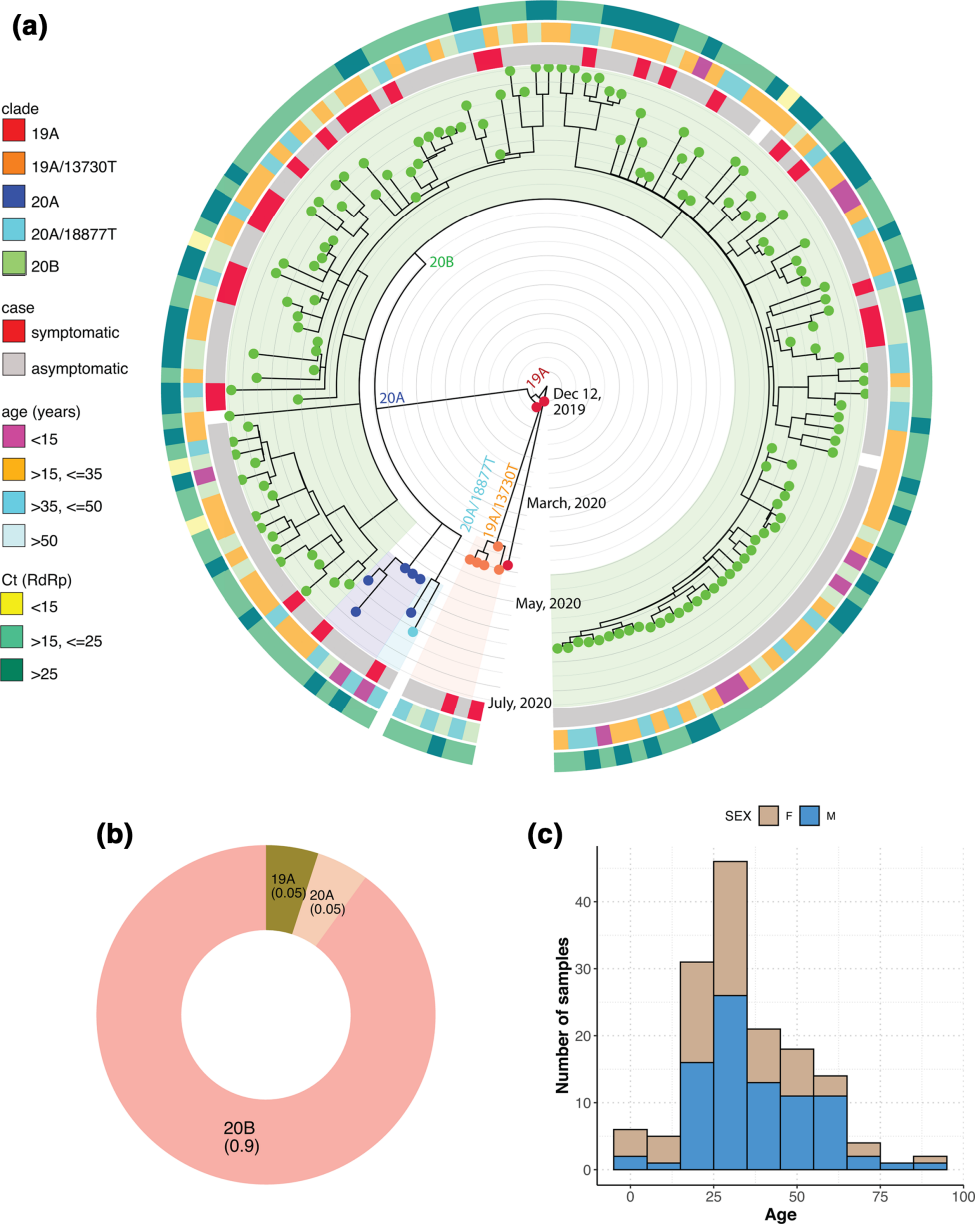
Phylodynamic tree of the samples as analysed using Nextstrain (a). Time-resolved phylogenetic tree from Nextstrain analysis; (b) distribution of clades across the dataset; (c) symptoms vs case distribution for samples classified as belonging to 20B clade.

The phylogenetic tree was rooted with respect to the sequence from the Wuhan Wu-1 strain (NC_045512.1), which has been annotated as the base clade 19A. One sample (collected in April 2020) seemed to directly emerge from this base clade (Fig. 2a). Further, five samples belonged to the 19A clade but also harboured the C13730T mutation (establishing sub-clade 19A/C13730T). The C13730T mutation has been previously reported to be associated with a more virulent variant of the strain [23]. However, we did not detect the C13730T mutation in the remaining dataset. The 19A/C13730T subclade was marked by the presence of missense mutations G1820A (ORF1A, nsp2, G519S), C6310A (ORF1a, nsp3, S2015R), C6312A (nsp3, T2016K), C28311T (N, P13L) and two synonymous mutations C19524T (nsp14, L6425L) and C23929T (S, Y789Y) (all amino acid numbering correspond to ORF1a/1ab polyprotein chain). Since only five samples were classified as 19A in our dataset, we hypothesized that this strain eventually faded out from the population from June onwards. The first major split in our dataset was observed during the beginning of April, marking the appearance of the 20A clade. The 20A clade was characterized by the presence of co-occurring mutations C241T (5′-UTR), synonymous C3037T (nsp3, F924F), and missense C14408T (nsp12, P4720L) and A23403G (S, D614G) (Figs 2a and S3). This follows the global trends where the S protein mutation D614G has been identified to be associated with the most prevalent strain circulating worldwide. Seven samples belonged to the archetypical 20A clade, whose origin has been directly traced to the viral strains characterized during late February to March in European countries (Italy, Germany) [24]. From the middle of April and onwards, the distribution profile was strongly dominated by the 20B, which also formed the second cluster within this division, with a characteristic triplet mutation of G28881A, G28882A and G28883C in the N protein (Figs 2 and S3. Of all the clades identified, our dataset was strongly populated by the presence of a single major clade viz. 20B, evident from the single large cluster (shaded light green) in Fig. 2a. This single large cluster seemed to arise independently from the cluster defined by 19A/C13730T subclade, possibly indicating two independent modes/routes of viral introduction in the local population. Overall, the phylodynamic clustering of samples suggested the presence of two minor (19A-5%, 20A-5%) and one prevalent, major clade (20B-90%) (Fig. 2b). We also performed Nextstrain analysis separately on our Illumina and Nanopore datasets and observed that the clusters obtained did not show any segregation due to the platform used and were distributed across the clades (data not shown), thus ruling out the possibility of incorporation of any bias due to the sequencing method used.

When the clade distribution was overlaid with different clinical features, the 19A clade contained samples belonging to age groups >35 years old, with moderate Ct values (>15, <=25 Ct). Within the dominant 20B clade, a major proportion of samples were aged between 15 and 50 years and were asymptomatic (Fig. 2a, c), while the symptomatic patients mainly belonged to high-risk age group (>50 years). As already mentioned above, exceptionally low viral Ct values (<15) were found to be almost always associated with asymptomatic samples (three of four samples in Fig. 2a, data N.A. for one sample), while the association of moderately low Ct values (>15, <=25) also appeared to be biased towards asymptomatic patients. This bias could be explained partially, if we note the presence of a single, close knit cluster (located in the extreme right of the phylodynamic tree (Fig. 2), where all the samples were classified as asymptomatic, during sample collection. Interestingly, this cluster consisted of samples that were collected from a single suburban neighbourhood located just outside Hyderabad (Fig. S4). This specific group of samples consisted of patients with mixed age groups, albeit with little correlation between age and viral load.

### Mutational landscape of SARS-CoV-2

**Fig. 3.**
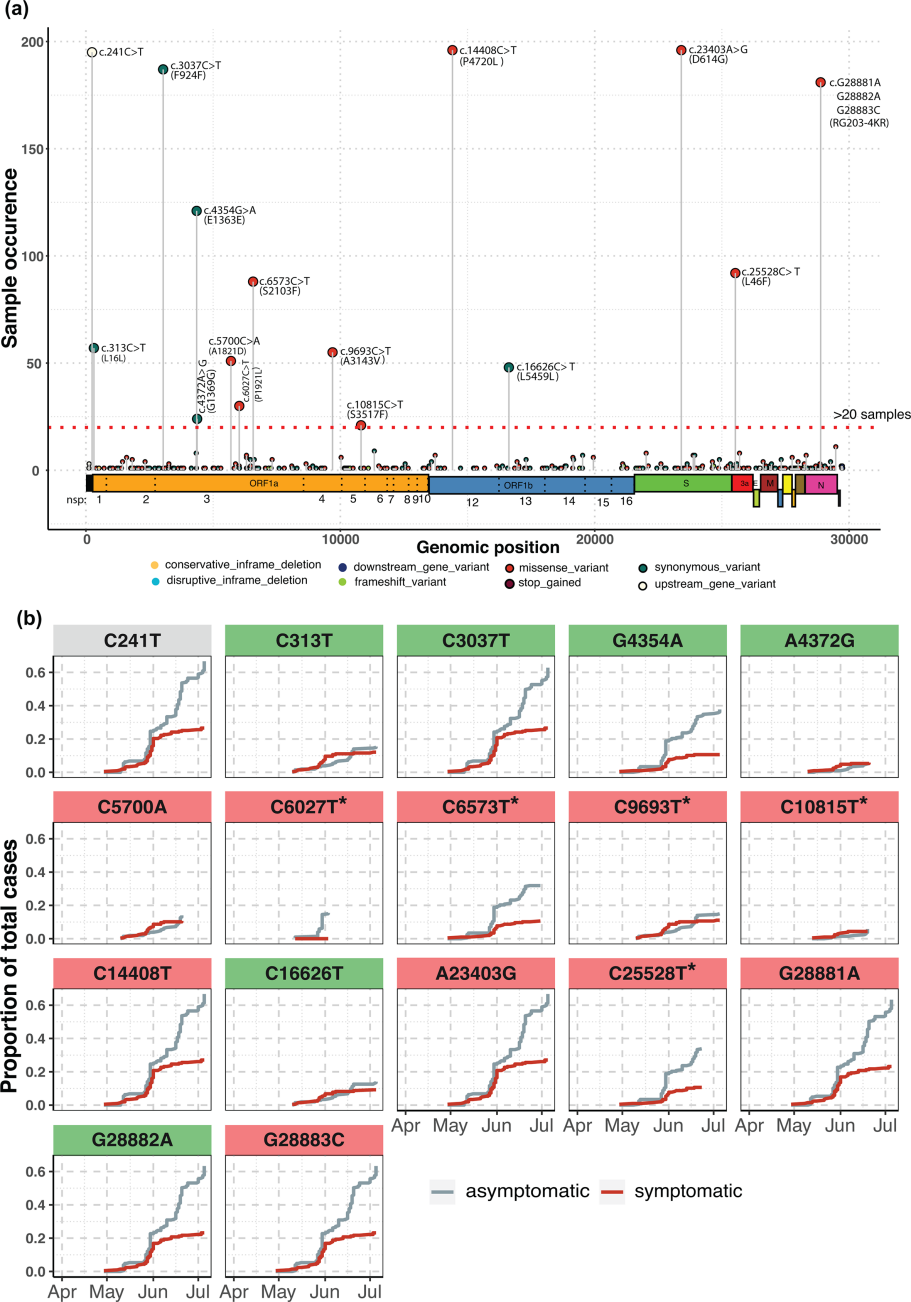
Distribution of all high-frequency mutations as called on Illumina and Nanopore sequencing data. (a) Genomic locations of all high-frequency mutations (samples >10 %, indicated by red, dashed horizontal line); (b) cumulative distribution of symptomatic and asymptomatic cases for each of the 17 high-frequency mutations. The panels for missense and synonymous mutations are highlighted in red and green shades, respectively. The unique mutations in nsp3, nsp4, nsp5 and ORF3a are indicated with a star.

From the mutation analysis on the filtered, combined pool of 207 sequences, we obtained a total 301 mutations across the SARS-CoV-2 genome (Data S1). Of the 301 mutations, 17 were present in >10 % of samples (Fig. 3a). Of these 17 high-frequency mutations, seven viz. C241T (5′-UTR), C3037T (nsp3, ORF1a), C14408T (nsp12, ORF1ab), A23403G (S), G28881A, G28882A, G28883C (N) were highly recurrent (present in >80 % of the samples; Fig. 3a). The A23403G (D614G) mutation in Spike protein was identified in samples as early as the beginning of April 2020. Although a highly recurrent mutation in multiple demographics, no clear correlation has been established between D614G mutation and severity of disease [24–26]. The D614G mutation has almost invariably been found to be associated with C241 >T, C3037 >T (a silent mutation) and a mutation in nsp12 (RdRp) C14408 >T as reported [27]. The haplotype defined by the co-occurrence of these four mutations is the current dominant form of SARS-CoV-2 virus circulating across the world [28–30] (https://www.gisaid.org/epiflu-applications/phylodynamics/).

Apart from these four mutations, we also observed moderate to high frequencies of other mutations, especially in the nsp3 protein encoded by ORF1a viz. G4354A, A4372G, C5700A, C6027T, C6573T, the latter three being missense mutations. Of these, C5700 along with a silent C313T mutation has been reported to co-occur in samples collected from the Western state of Maharashtra, India [31]. The nsp4 and nsp5 proteins each harboured one high-frequency missense mutation each namely C9693T and C10815T, respectively. Similarly, the ORF3a region possessed one high-frequency missense mutation C25528T. Further, for each of the 17 high-frequency mutations identified in this set, we calculated the cumulative increment in the frequency of case symptoms as a function of time and noted a consistent increase in the frequency of asymptomatic cases specially for missense mutations (Fig. 3b). Seven of the seventeen mutations, including the signature A23403G-associated mutations, were associated with a higher fraction of asymptomatic cases, with trends indicating towards a consistent rise in asymptomatic cases, towards the end of the sample-collection timeline (Fig. 3b).

**Fig. 4.**
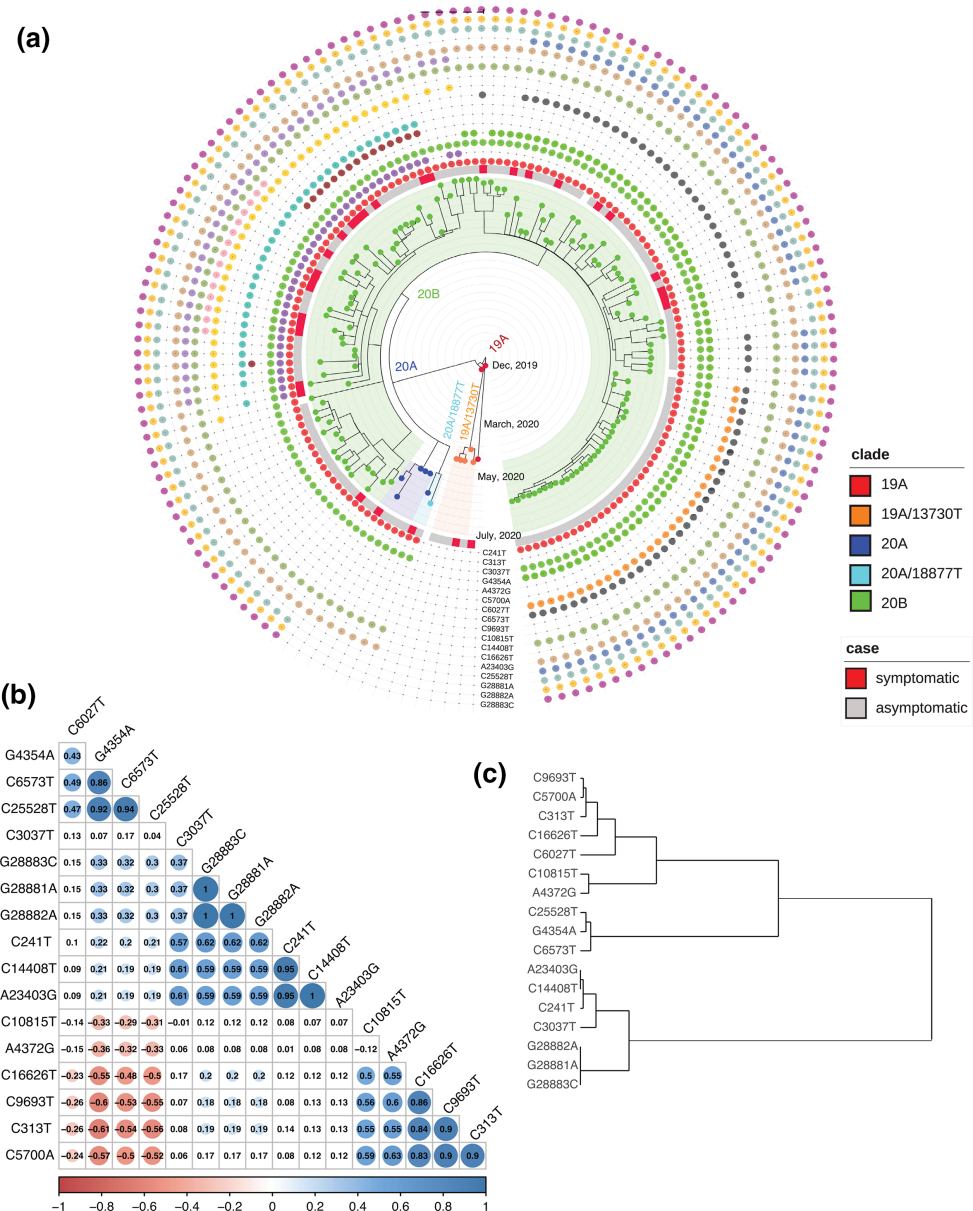
(a) Mapping of the 17 high-frequency mutations on the phylodynamic tree calculated using Nextstrain; (b) pairwise correlation coefficient matrix for the aforementioned mutation set; the numbers within the cells indicate *P*-values; (c) associated dendrogram obtained after hierarchical clustering of mutations.

When we mapped the high-frequency mutations (Fig. 4a) on the phylodynamic tree to investigate whether the mutational spread of all high-frequency mutations (enumerated in Fig. 3a) was consistent across the clusters belonging to different nodes, or whether there existed any mutual exclusivity between the nodes on the basis of these variations, we could discern the presence of two broad subgroups of mutations within the 20B clade (Fig. 4a). While the entire 20B clade was defined by the presence of the well-established, canonical signature mutations viz. C241T, C3037T, C14408T, A23403G, and G28881A, G28882A, G28883C triplet variant, other variants were dispersed in a more mutually exclusive manner across the samples. The correlation coefficient matrix (Fig. 4b) combined with hierarchical clustering (Fig. 4c) suggested a positive association between the nsp3 mutations C4354A and C6573T and the ORF3a mutation C25528T, which can also be seen in terms of similar sample occurrence (Fig. 4a). These three variants potentially constituted one branch within the 20B clade, comprising approximately 90–125 samples (43–60 % of the dataset). There was a low to moderately negative correlation between the set defined by these three mutations and another set defined by C10815T (nsp5), A4372G (nsp3) (low) and C16626T (nsp13), C9693T (nsp4), C313T (nsp1) and C5700A (nsp3) (moderate). The latter three potentially giving rise to another major branch within 20B, comprising 51–57 samples (24–27 % of the dataset). It was interesting to note the presence of mutual exclusivity between mutations identified within the nsp3 gene (Fig. 4a–c).

In order to uncover the dynamic landscape of variation in the SARS-CoV-2 genome present in the population pool under this study, we additionally evaluated low-frequency mutations (Fig. S5a, b). This becomes important since some of these low-frequency mutations might influence viral life cycle and disease progression, imparting drug resistance to a small subset of host population, and identifying variability in the selection pressures on different genomic regions [32]. The analysis indicated that certain regions appeared to be vulnerable to harbouring low-frequency mutations. Specifically, the nsp3 regions c.5700–6573, and c.27894–28470 (encompassing the region between ORF8 and N proteins) showed the presence of dense clusters of low-frequency mutations (Fig. S5a, b). From an estimated mutation rate (per base per sample) calculated for each gene in this dataset (Table S1), we identified the highest rate of variations in the region encoding nsps 3, 5, 8 and 14 among all the non-structural proteins. Among the major structural proteins, nucleocapsid (N) had the highest mutation rate. A majority of the samples investigated in this work carried approximately 10–15 mutations per sample (Fig. S6). To the best of our knowledge, this is the first report on low-frequency SARS-CoV-2 mutations from India.

### Phenotypic implications of mutations in non-structural and accessory proteins

Of all the high-frequency mutations described in earlier sections, nine were missense mutations, and were localized to nsp3, nsp4, nsp5, nsp12, S, ORF3a and N proteins. Our analysis also highlighted the presence of unique nsp3 (C6027T, P1921L; C6573T, S2103F), nsp4 (C9693T, A3143V) and ORF3a (C25528T, L46F) mutations specific to our dataset, which has not been reported elsewhere to date. While the effect of spike D614G (A23403G) and nsp12 P323L (C14408T) mutations on the corresponding protein functions have been studied extensively [24–27, 33], we endeavoured to analyse the effect of missense mutations in nsp3, nsp4 and ORF3a. Nsp3 is the largest of all non-structural coronaviral proteins and acts as a scaffold for formation of the replication-transcription complex (RTC) by forming multi-subunit assemblies with other non-structural proteins. Nsp3 has also been shown to be involved in the formation of nuclear pore complexes, which span the double membrane vesicles of replication organelles (RO), which form near the transformed endoplasmic reticulum in viral-infected cells [34]. The domain architecture of nsp3 reveals that the recurrent C5700A (A1812D) mutation lies in the crucial viral protease domain, while the novel C6027T (P1921L) lies in the nucleic acid binding region (NAR). No association of C6573T with any functional domain was however found (Fig. S7a). As per the structural model of SARS-CoV-2 nsp3 viral protease domain (PDB- 6W9C), the A1812D mutation has the potential to modify the surface electrostatic environment in the vicinity of protease domain (Figu. S7b). In the absence of a full-length nsp3 structure, however, it is difficult to predict how this locally modified surface potential will affect interaction between neighbouring domains, as well as how C6027T mutation lying in the NAR domain might alter domain architecture. A single high-frequency nsp4 mutation C9693T (A380V) lay in a region proximal to a putative transmembrane domain (Fig. S7c). The 3a mutation C25528T (L46F) lay at the terminus of the first transmembrane spanning helix (Fig. S7d).

## Discussion

In the current study, we have presented a comprehensive map of the mutations identified from the confirmed COVID-19 cases collected from the southern state of India, Telangana. After a slow progress of the outbreak during the months of February–April, the state has been witnessing a constant upsurge in the number of infections and has been listed as one of the highly affected states in the country. The data suggested a positive association between asymptomatic cases and lower Ct values (or higher viral load), which was a surprising observation in contrast to report from a previous study carried out on samples collected predominantly from a western state of the country [31]. However, such correlations have also been reported earlier [35]. Another study reported that the SARS-CoV-2 viral load may peak prior to symptom onset [36] providing a possible explanation for our observations. More importantly, our results educate us to be wary of the possibility of viral spread in the community through such 'hidden' infections [37] .

Identifying the mutations from samples collected over a period of time, provides a way to assess the genomic evolution, which the virus might have undergone during infection and transmission. With these aspects in our purview, we have attempted to characterize the genomic epidemiology of SARS-CoV-2 and a comprehensive mutational landscape, using a dataset of 210 samples sequenced using both Illumina and Nanopore sequencing technologies. The phylodynamic analysis revealed that a majority of our samples belonged to the 20B clade, with the clade seeming to appear in the beginning of April and then dominated the sample profiles later on. In the absence of detailed travel histories, it is difficult to establish a direct link between the clades' classification and the source of strain introduction in the population.

More importantly, mutational analysis revealed the presence of unique mutations in the samples from Telangana, especially in nsp3, nsp4, nsp5 and ORF3a. The SARS coronaviruses nsp3 is a papain-like protease (PLP2) and nsp5 is a 3C-like protease (3CLpro) also called as main protease (M^pro^), both required for cleavage of polyproteins pp1a and pp1ab to generate 16 non-structural proteins (nsp1-16) [38]. Nsp3 is also the largest multi-domain protein encoded by SARS-CoV-2 and other coronaviruses [39]. The mutations identified in nsp3 viz. C5700A (A1812D) and C6027T (P1921L) lie in the main viral protease like (analogous to PLP2 in SARS-CoV) and nucleic acid binding (NAR) domains, respectively [39]. While C6573T was not directly associated with any major catalytic domain in SARS-CoV-2, the nucleotides 5818–6975 in highly similar SARS-CoV, form what is known as extended papain like protease domain (papain like non-canonical (PLnc)) of the protein. The PLnc domain in SARS-CoV has been shown to interact with a large number of nsp partners during viral replication and transcription cycle and acts as a scaffold for membrane-associated replication/transcription complex (RTC) formation. The formation of RTC also requires nsp3 association with nsp4 and nsp5 [40]. Hence, it can be speculated that these mutations may alter the balance or stoichiometry of the complex formation, perhaps causing viral RTC reprogramming. The RTC complexes in coronaviruses have been shown to perform multiple tasks during replication and transcription by being able to form compositionally diverse complexes, each specific to carry out a particular process. It is possible that these mutations could bring about a switch in this compositional programming [19].

SARS-CoV-2 ORF3a encodes an accessory, transmembrane, ion-channel protein, belonging to the class of proteins called Viroporins, which induce innate immune signalling receptor NLRP3, leading to production of cytokines IL-1β, IL-6 and tumour necrosis factor (TNF) [41]. This often results in tissue inflammation in a SARS-CoV-2 infection. The most common mutations identified in ORF3a among the prevalent lineages across the world is Q57H and G251V [42]. However, the C25528T (L46F) mutation observed in this study localizes to the domain I in the N-terminal signal-peptide region of ORF3a, similar to Q57H mutation. This region is critical for viral localization to the Golgi apparatus. This single, highly recurrent 3a variation found in our 20B cluster is different from the most common 3a variants identified in other parts of the country where G25563T/Q57H is the most preponderant form [43]. Functional impact of these mutations on the location, and subsequent translation and assembly, requires additional work.

The effect of the mutations detected in this study on the eventual protein function constitute a critical topic of interest due to the direct implications it might have on the design of antiviral vaccines and therapeutics. However, given that the SARS-CoV-2 genome is ultimately a single-stranded RNA chain, which like other viral RNA genomes [44, 45], has been shown to fold into multiple independent, modular domains [46, 47], the probable effect of the mutations on RNA secondary structure should also be evaluated. Interestingly, several of our mutations especially the ones in nsp3, were found to coincide with highly structured regions. It would be interesting to pursue, from an academic point of view, whether any of these mutations affect the local secondary structure and stability of the genome, thereby affecting RNA–protein interactions, which play a role in nearly all the stages of the viral life cycle.

In conclusion, we report the comprehensive mutation landscape of more than 200 individual SARS-CoV-2 viral genome sequences isolated from COVID-19 patients or primary contacts. The data forms a starting point for the state government machinery to conduct further studies on virus transmission helping in taking informed public health decisions. The genomic mutations also inform on potential mechanisms being employed during evasion of the host’s immune response and traces of higher or lower pathogenicity, if any, being developed over the span of time, thus significantly impacting efforts in vaccine development.

### Data availability

The consensus FASTA sequences of all samples have been provided in Data S3. All raw sequencing data used in this study have been submitted to the Sequencing Read Archive (SRA) with project accession ID PRJNA691556

(http://www.ncbi.nlm.nih.gov/bioproject/691556). In addition, sequences for 135 samples have been submitted to GISAID databases. Their accession IDs have been provided in Data S4.

## Supplementary Data

Supplementary material 1

Supplementary material 2
