# Supplementary material for: A comprehensive profile of genomic variations in the SARS-CoV-2 isolates from the state of Telangana, India
Source: J Gen Virol. 2021 Feb 15;102(3):001562. doi: 10.1099/jgv.0.001562 (PMC8515869; doi:10.1099/jgv.0.001562)
Supplement: Supplementary material 1 [file jgv-102-1562-s001.pdf]

Supplementary data

Supplementary figure S1: Detailed workflow of the methodology and various stages of the analysis adopted in this study.

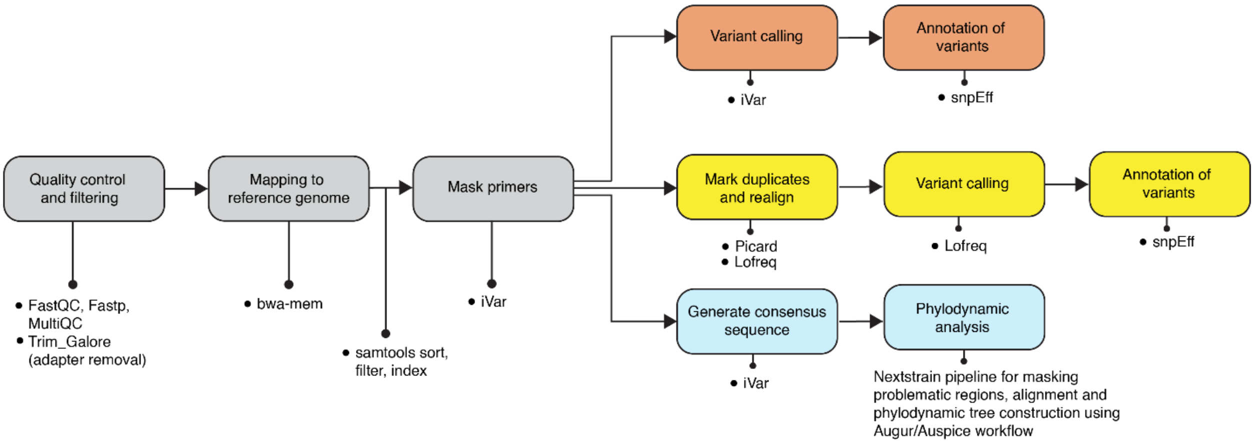

Supplementary figure S2: Sample features: (A) Ct value distribution of samples with respect to date of sample collection; (B) Correlation between age and Ct values of the samples; (C) Relation of symptoms with age, p-value estimated using Wilcoxon rank-sum test at .05 significance level(D) Distribution of samples with respect to locations within Telangana.

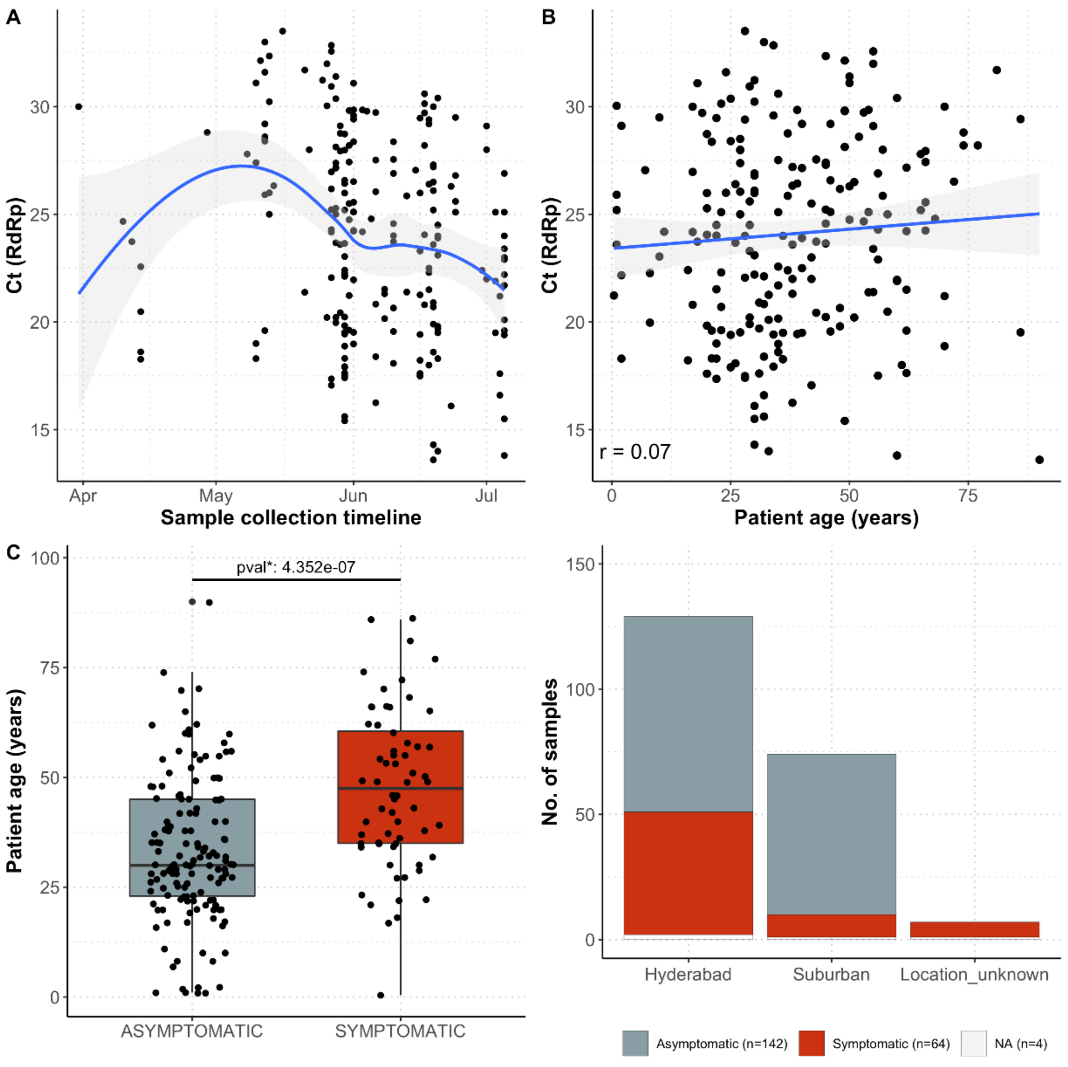

Supplementary figure S3: Time resolved phylogenetic tree created using Nextstrain, indicating major mutations marking the major branch points:- A: ORF1b (P314L), S (D614G); B: N (R203K, G204R), ORF14 (G50N); C: ORF1a (A1812D); D: ORF1a (A3143V); E: N (D401Y), ORF1a (S3517F); F: ORF1a (S2103F); G: ORF3a (L46F); H: ORF1a (P1921L).

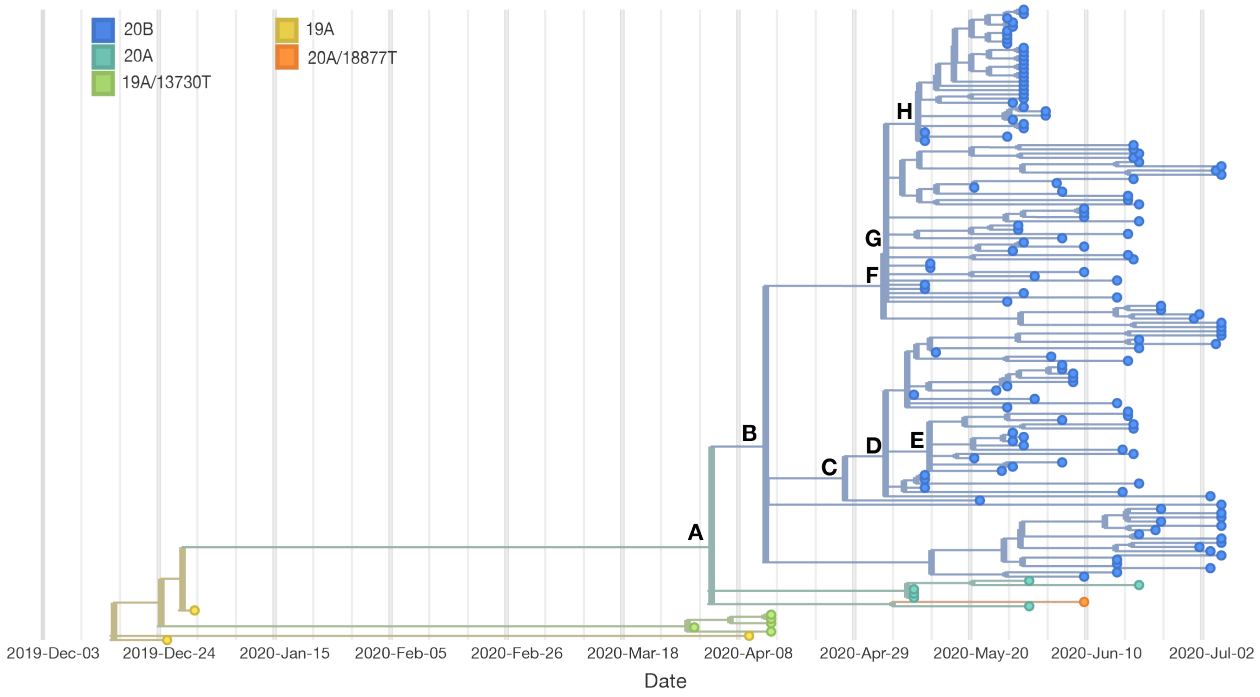

Supplementary figure S4: (A) Phylogenetic tree indicating local distribution of samples within and outside Hyderabad. (B) Detailed lineage assignment by pangolin on the dataset

(A)

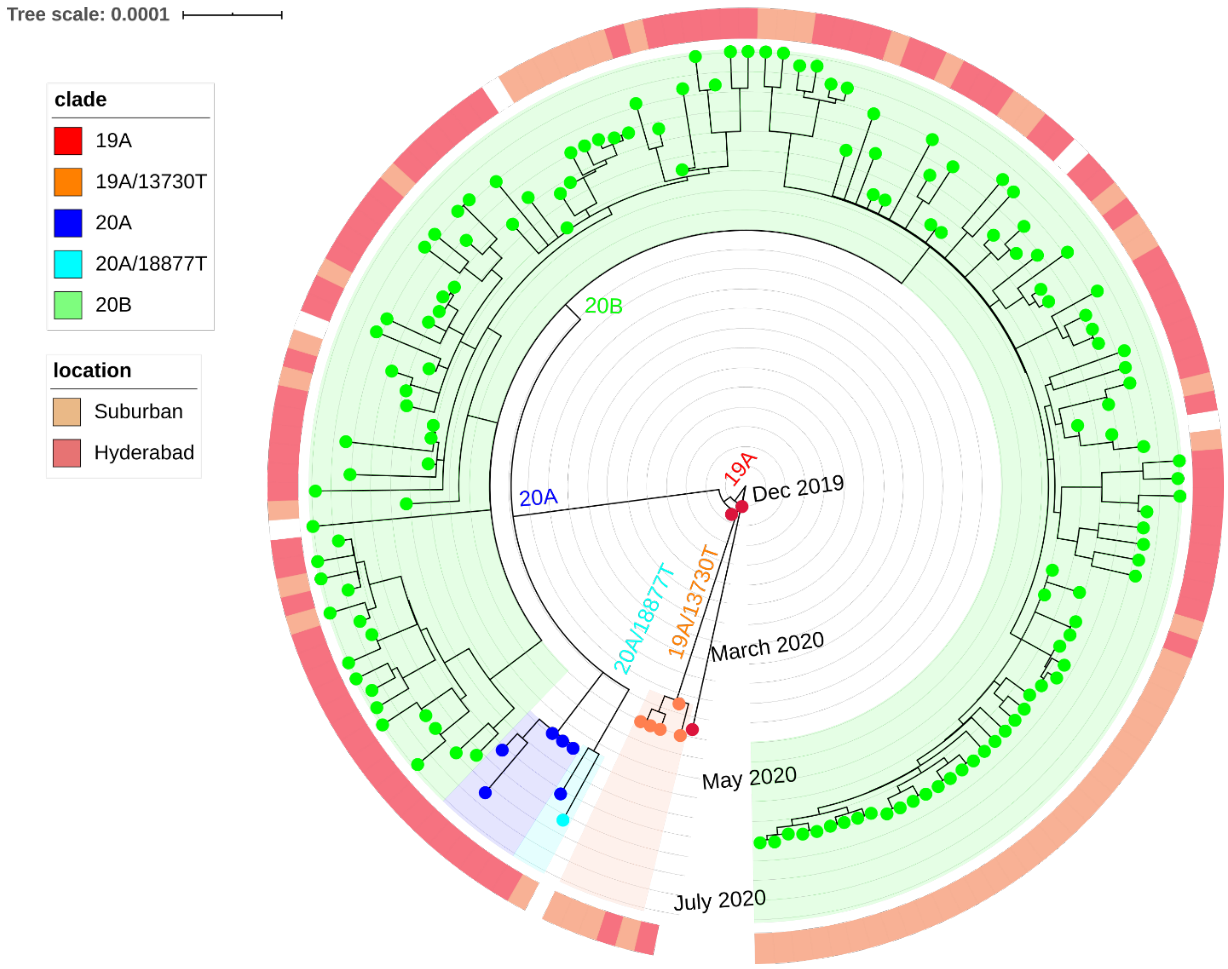

(B)

	lineage	Number of samples	Earliest date	Most common countries
1	B.1.1.8	92	May 11, 2020	India (98%), UK(2%)
2	B.1.1.32	53	March 24, 2020	India (94%), UK(4%), USA(2%)
3	B.1.1	44	February 10, 2020	UK (83%), USA (5%), Portugal (1%)
4	B.1	11	January 10, 2020	UK (49%), USA (29%,) France (2%)
5	B.6	7	February 14, 2020	India (41%), Singapore(40%), Australia (5%)
6	B.1.36	2	February 16, 2020	UK (47%), India (25%), Saudi_Arabia (12%)
7	B	1	December 24, 2020	UK (55%), China (15%), USA (11%)
8	B.1.1.46	1	April 20, 2020	India (76%), UK (15%), Brazil (4%)
9	B.1.1.72	1	March 28, 2020	UK (41%), India (37%), Singapore (14%)
10	B.1.26	1	March 15, 2020	USA (89%), UK (5%), New_Zealand (3%)
11	B.1.5	1	February 26, 2020	UK (66%), USA (12%), Spain (12%)
12	B.4	1	January 18, 2020	UK (20%), Australia (17%,) India (12%)

Supplementary figure S5: (A) Low frequency mutations (<20 & >1 sample) across the dataset; (B) Lofreq identified low frequency mutations. Orange line segments indicate mutations with low allele frequency (<0.25). A detailed amino acid nomenclature for each mutation is provided in the supplementary data sheets D1 and D2.

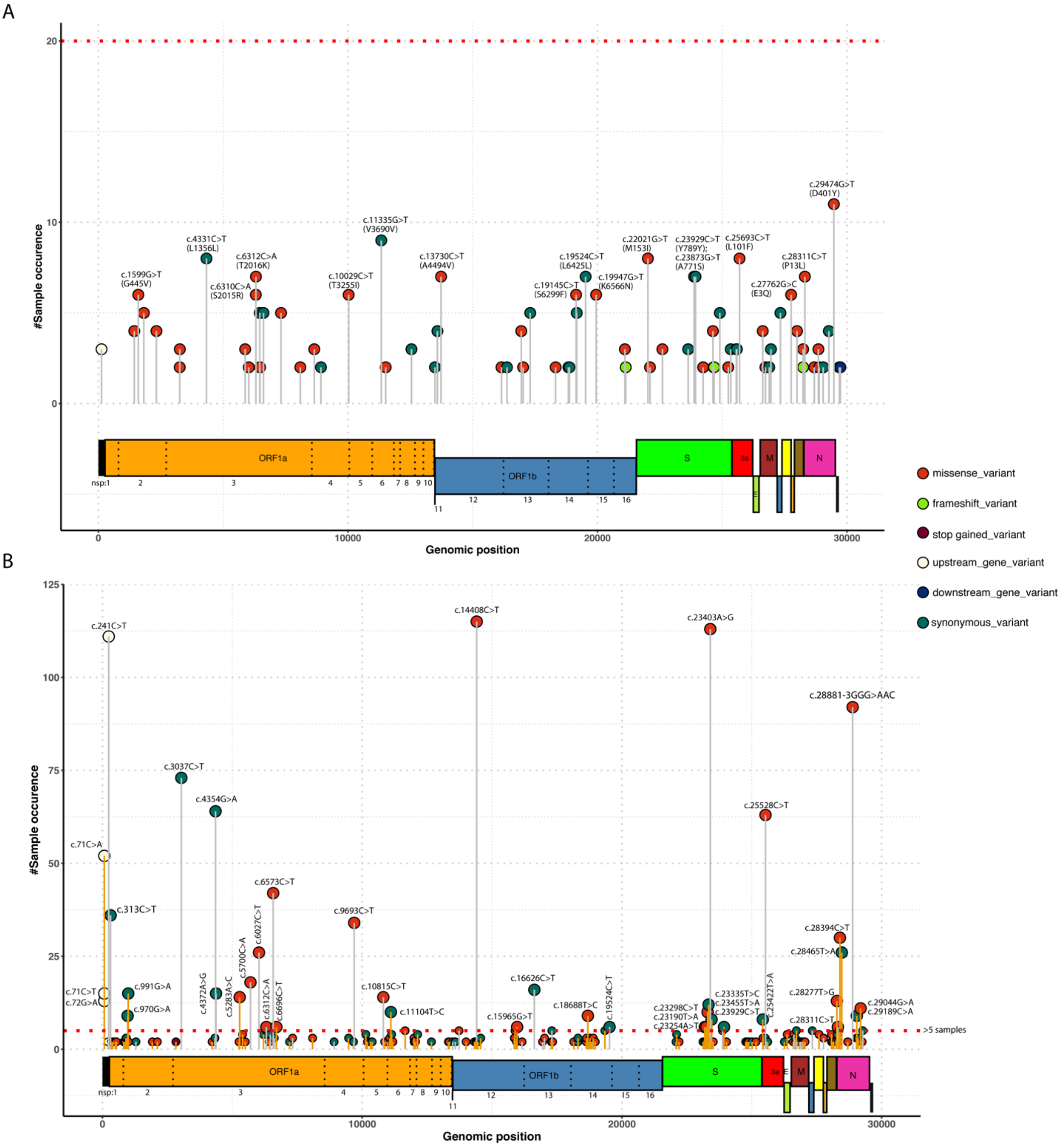

Supplementary figure S6: Distribution of unique mutations per sample in the dataset.

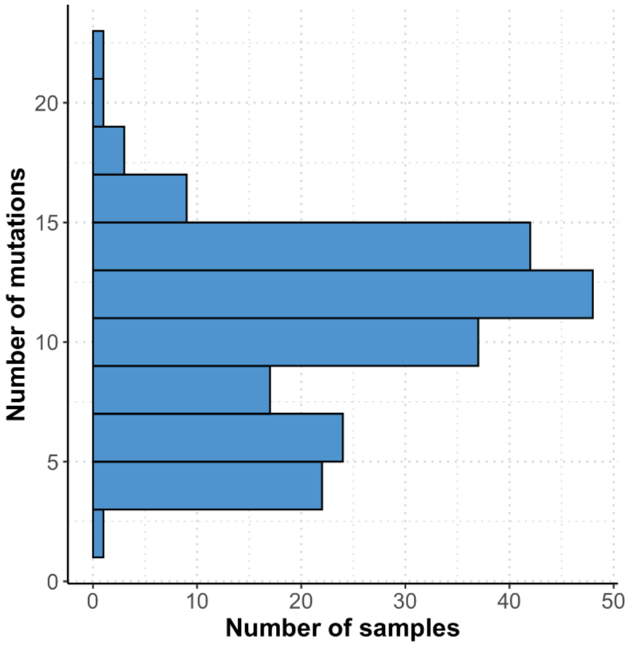

Supplementary figure S7: Domain architecture of nsp3, nsp4, nsp5 and ORF3a proteins (adapted from NCBI RefSeq). (A) nsp3 (B) structural model of nsp3 protease domain (PDB-6W9Y) with Ala1812Asp mutation highlighted (Ala249Asp) as per amino acid numbering initiated from only protease domain solved in this structure. The bottom panel shows the surface representation of electrostatic potential as calculated from Adaptive Poisson Boltzmann solver (APBS)[42] (C) nsp4 (D) ORF3a

(A)

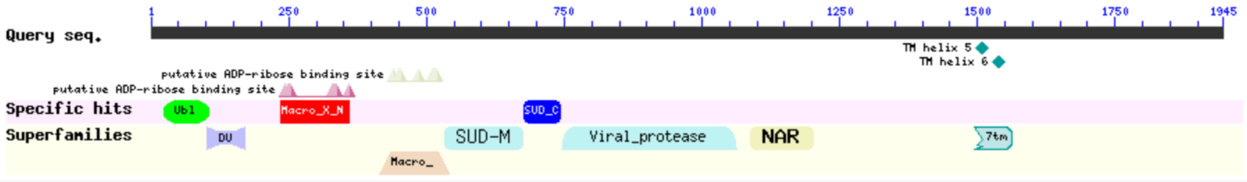

(B)

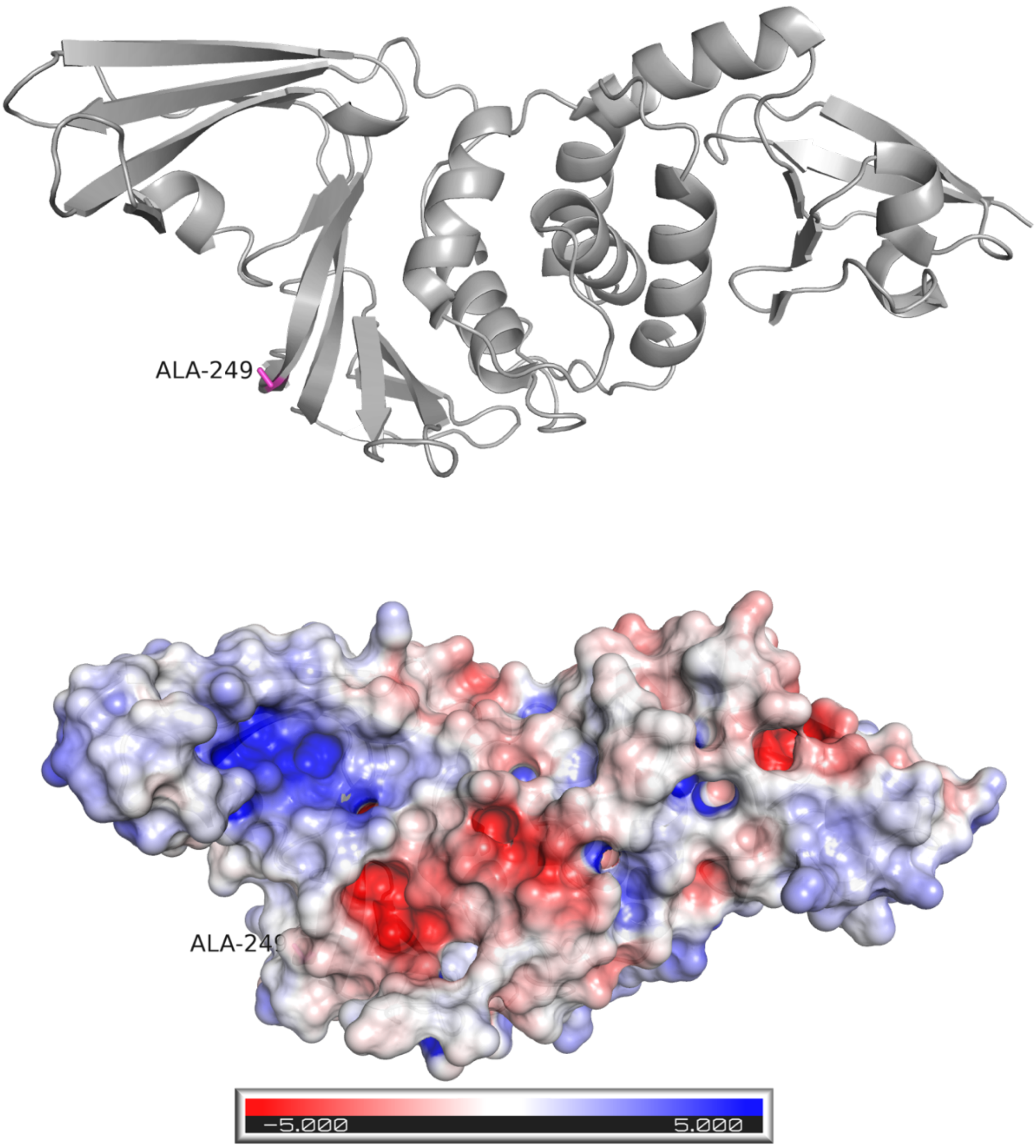

(C)

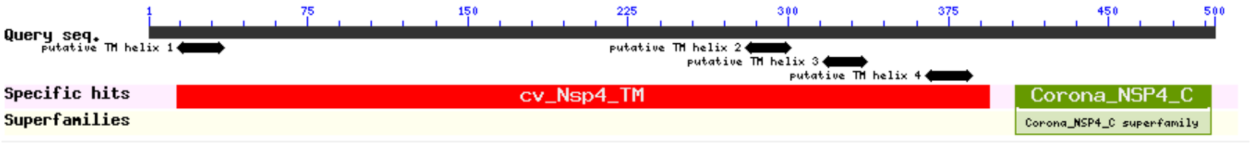
Query seq.
putative TM helix 1
putative TM helix 2
putative TM helix 3
putative TM helix 4
Specific hits
cv_Nsp4_TM
Corona_NSP4_C
Superfamilies
Corona_NSP4_C superfamily

(D)

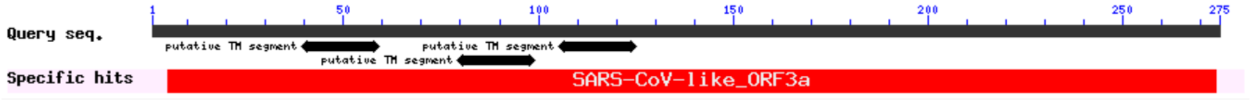
Query seq.
putative TM segment
putative TM segment
putative TM segment
Specific hits
SARS-CoV-like_ORF3a

Supplementary Table 1: SARS-CoV-2 gene lengths and mutations identified per bp per sample. nt indicates nucleotide.

SARS-CoV-2 genes	Genomic positions	Mutation per bp per sample
nsp1	nt. 266-805	$4.4x10^{-5}$
nsp2	nt. 806-2719	$5.2x10^{-5}$
nsp3	nt. 2720-8554	$4.5x10^{-5}$
nsp4	nt. 8555-10054	$3.2x10^{-5}$
nsp5	nt. 10055-10972	$5.7x10^{-5}$
nsp6	nt. 10973-11842	$3.3x10^{-5}$
nsp7	nt. 11843-12091	$1.9x10^{-5}$
nsp8	nt. 12092-12685	$4.8x10^{-5}$
nsp9	nt. 12686-13024	0
nsp10	nt. 13025-13441	$2.3x10^{-5}$
nsp12	nt. 13442-16236	$2.6x10^{-5}$
nsp13	nt. 16237-18039	$4.3x10^{-5}$
nsp14	nt. 18040-19620	$4.9x10^{-5}$
nsp15	nt. 19621-20658	$4.3x10^{-6}$
nsp16	nt. 20659-21552	$3.2x10^{-5}$
S	nt. 21563-25384	$5.1x10^{-5}$
ORF3a	nt. 25393-26220	$9.6x10^{-5}$
E	nt. 26245-26472	$4.2x10^{-5}$
M	nt. 26523-27191	$7.9x10^{-5}$
ORF6	nt. 27202-27387	$7.7x10^{-5}$
ORF7a	nt. 27394-27759	$6.5x10^{-5}$
ORF7b	nt. 27756-27887	$1.1x10^{-4}$
ORF8	nt. 27894-28259	$1.3x10^{-4}$
N	nt. 28274-29533	$1.01x10^{-4}$

Additional supplementary data:

1. Supplementary data D1: List of all mutations identified across all samples in the iVar based analysis (provided separately as the first worksheet titled 'Supplementary_data_D1_ivar_call' in excel file ‘Supplementary_data_sheets.xlsx).
2. Supplementary data D2: List of all mutations identified across all samples in the lofreq based analysis (provided separately as the second worksheet titled 'Supplementary_data_D2_lofreq_ca' in excel file ‘Supplementary_data_sheets.xlsx).
3. Supplementary data D3: Consequence sequence of 207 individual patient viral genomes analysed (provided separately as the third worksheet titled 'Supplementary_data_D3_Cons_seq' in excel file ‘Supplementary_data_sheets.xlsx).
4. Supplementary data D4: Accession IDs for 135 sequences submitted in GISAID Database (provided separately as the fourth worksheet titled 'Supplementary_data_D4_GISAID_AC' in excel file ‘Supplementary_data_sheets.xlsx).
